# Effects of Milk-Derived Extracellular Vesicles on the Colonic Transcriptome and Proteome in Murine Model

**DOI:** 10.3390/nu14153057

**Published:** 2022-07-26

**Authors:** Chunmei Du, Yiguang Zhao, Kun Wang, Xuemei Nan, Ruipeng Chen, Benhai Xiong

**Affiliations:** State Key Laboratory of Animal Nutrition, Institute of Animal Sciences, Chinese Academy of Agricultural Sciences, Beijing 100193, China; duchunmeim@163.com (C.D.); zhaoyiguang@126.com (Y.Z.); cang327@163.com (K.W.); xuemeinan@126.com (X.N.); chen_ruipeng@yeah.net (R.C.)

**Keywords:** inflammatory bowel disease, milk extracellular vesicles, genes, proteins

## Abstract

Evidence shows that effective nutritional intervention can prevent or mitigate the risk and morbidity of inflammatory bowel disease (IBD). Bovine milk extracellular vesicles (mEVs), a major bioactive constituent of milk, play an important role in maintaining intestinal health. The aims of this study were to assess the effects of mEV pre-supplementation on the colonic transcriptome and proteome in dextran sulphate sodium (DSS)-induced acute colitis, in order to understand the underlying molecular mechanisms of mEV protection against acute colitis. Our results revealed that dietary mEV supplementation alleviated the severity of acute colitis, as evidenced by the reduced disease activity index scores, histological damage, and infiltration of inflammatory cells. In addition, transcriptome profiling analysis found that oral mEVs significantly reduced the expression of pro-inflammatory cytokines (*IL-1β*, *IL-6*, *IL-17A* and *IL-33*), chemokine ligands (*CXCL1*, *CXCL2*, *CXCL3*, *CXCL5*, *CCL3* and *CCL11*) and chemokine receptors (*CXCR2* and *CCR3*). Moreover, oral mEVs up-regulated 109 proteins and down-regulated 150 proteins in the DSS-induced murine model, which were involved in modulating amino acid metabolism and lipid metabolism. Collectively, this study might provide new insights for identifying potential targets for the therapeutic effects of mEVs on colitis.

## 1. Introduction

Inflammatory bowel disease (IBD) is an incurable inflammatory disorder, which impairs the quality of life of patients and creates substantial costs for health care [1]. Moreover, IBD has evolved into a global disease with increasing incidence in every country [1]. The mechanisms of IBD pathogenesis are the result of an interaction between genetic predisposition, environmental factors, intestinal barrier and immune imbalance [2]. Genetic predisposition explains about 25% of the overall incidence of IBD, and environment factors play an increasingly considerable role in the development of IBD [3,4]. Therefore, manipulation of the susceptibility of gene expression via dietary intervention might help to reduce the risk of IBD [5,6].

Recently, dietary supplements or functional ingredients have gained wide attention, due to their nutritional and therapeutic values, as well as minimal side effects, even after long-term consumption. Milk extracellular vesicles (mEVs), with biomolecular lipid bilayer nanostructures and various cargos, are abundant bioactive ingredients in bovine milk [7]. Thousands of components of various types have been identified in mEVs, including predominantly proteins, nucleic acids, and lipids [8]. Among these cargos, miRNA, lncRNA and circRNA have been demonstrated to participate in regulating a wide variety of gene expression and multiple processes in hosts [9]. For example, Gao et al. found that mEV-derived bta-miR-34a could activate the apoptosis signaling pathway, thus improving intestinal epithelial cell survival [10]. Moreover, emerging evidence supports the claim that mEVs could enhance intestinal immunity [11], attenuate intestinal epithelial cell death [12], and exert protective effects against oxidative stress [13]. There is also a previous study of the effect of mEV supplementation on the gene expression of chronic experimental colitis [14]. However, the study of the protective effects of mEVs on acute colitis has focused mainly on phenotype changes [15], which may have restricted the knowledge and in-depth study of the roles of mEVs on the genes and protein expression mechanisms behind the attenuation of acute colitis.

The applications of multi-omics approaches to identify targets for therapeutic interventions are high on the research agenda for various diseases and help find low abundance targets that were underrepresented before [16]. To date, multi-omics sequencing has been widely employed in investigating the effects of functional foods supplementation on colitis [17,18]. Therefore, in this study, gut transcriptome and proteome were adopted to characterize the effect of mEVs on acute colitis. In addition, the relationship between colonic gene expression and protein levels were also identified. This study not only enriches the biological function of mEVs, but may also uncover the potential mechanisms of mEVs that alleviate acute colitis and the underlying potential therapeutic targets for the treatment of IBD.

## 2. Materials and Methods

### 2.1. Milk Collection

Bovine raw milk samples were collected from mid-lactation Holstein cows at the farm of the Institute of Animal Science, Chinese Academy of Agricultural Sciences, Beijing, China. Then, the fresh milk samples were centrifuged immediately at 3000× *g* for 30 min at 4 °C to obtain whey. The whey samples were stored at −80 °C for further analysis.

### 2.2. mEVs Isolation from Whey

The whey samples were then performed as described previously to obtain mEVs [13,19]. Briefly, the samples were centrifuged at 12,000× *g* at 4 °C for 60 min to remove fat globules, casein aggregates and residual chymosin. The supernatant was then centrifuged at 30,000× *g*, 75,000× *g* for 60 min each to remove large particles and residual debris. The resulting supernatant was ultracentrifuged at 120,000× *g* for 90 min using an SW32Ti rotor (Optima XPN-100; Beckman Coulter Instruments, Fullerton, CA, USA). Finally, the pelleted mEVs were resuspended in PBS, filtered through 0.22 μm filters (Millipore, Burlington, MA, USA), and stored at −80 °C for further processing.

### 2.3. Western Blotting (WB)

Sodium dodecyl sulfate–polyacrylamide gel electrophoresis (SDS-PAGE) was used to separate equal protein amounts of mEVs and whey samples. Proteins were transferred onto polyvinylidene fluoride membranes, blocked with 5% skim milk (BD Biosciences, Franklin Lakes NJ, USA), and detected with the following primary antibodies: HSP70, TSG101, CD63, CD9 and calnexin (cat. no. ab275018, Abcam, UK). After incubation, the membranes were washed by TBST, and then incubated for 50 min at 37 °C with secondary antibody goat anti-rabbit IgG (ZSGB-bio, Beijing, China). The protein bands were washed by TBST and scanned with an automatic ECL image analysis system (Tanon-4800, Shanghai, China). In addition, the antibodies to verify globe proteome were ABAT (ab216465), SCIN (ab199723), DDC (ab131282), ACSL4 (ab155282) and GAPDH (ab9485), purchased from Abcam UK (Abcam, Cambridge, UK).

### 2.4. Transmission Electron Microscopy (TEM)

The isolated mEVs were stained with uranyl acetate and identified using a HT7700 transmission electron microscope (Hitachi, Tokyo, Japan).

### 2.5. Nanoparticle Tracking Analysis (NTA)

Recent reports support the claim that particle concentration might be more suitable to determine the levels of mEVs [20]. Hence, the particle size and concentration of mEVs were measured by NTA at VivaCell Shanghai with ZetaView PMX 110 (Particle Metrix, Meerbusch, Germany). Data were analyzed by the equipped ZetaView v8.04.02 SP2 software (Particle Metrix, Meerbusch, Germany).

### 2.6. Animals and Treatments

Six-week-old male specific pathogen-free (SPF) C57BL/6 mice were provided by SPF Biotechnology Co., Ltd (Beijing, China). All mice were adapted to the laboratory conditions with free access to food and water and housed at 22 ± 2 °C with 12 h light-dark cycles for two weeks. Animal care was performed following the guidelines for Care and Use of Laboratory Animals of the Chinese Academy of Agricultural Sciences and was approved by the Animal Ethics Committee of Chinese Academy of Agricultural Sciences (approval number: IAS2021-235).

Acute colitis was induced by the addition of 3.5% DSS (molecular weight 36,000–50,000 kDa; MP Biomedicals, CA, USA) to autoclaved drinking water for 7 days. Thirty-six mice were randomly divided into normal control (NC), DSS+PBS, and DSS+mEVs (3.0× 10^9^ particles per gram body weight) groups. Mice in the DSS+mEVs group received daily oral gavage with mEVs for 30 days, before addition of 3.5% DSS in the drinking water for 7 days. Body weight was monitored daily. Disease activity index (DAI) was determined by evaluating weight loss, stool consistency and presence of blood in the stool. All mice were sacrificed after anesthesia to measure the length of the colon. Part of the colon tissues was fixed in neutral buffered formalin and the rest of the samples were collected and stored at −80 °C for further analysis.

### 2.7. Histological Analysis

Fresh colon tissues were fixed in 10% neutral buffered formalin at 4 °C overnight. Paraffin was used to embed the tissues, which were then sliced to 10 μm thicknesses. Subsequently, the sections were stained with hematoxylin and eosin (H&E). Images of the sections were pictured using optical microscopy (Olympus, Tokyo, Japan).

### 2.8. Transcriptome Analysis

For RNA isolation, total RNA was extracted from the colon tissues using the mirVana™ miRNA Isolation Kit (Ambion, Austin, TX, USA) according to the manufacturer’s instructions. RNA integrity was evaluated using 2% agarose gel electrophoresis and an Agilent 2100 Bioanalyzer (Agilent, Santa Clara, CA, USA), and the purity of RNA was determined by NanoDrop 2000 (Thermo, Waltham, MA, USA). Only RNA integrity number (RIN) values above 7.0 were considered acceptable for sequencing.

RNA sequencing included the following steps: deplete Ribo-Zero and F=fragment RNA, synthesize first strand cDNA, synthesize second strand cDNA, purity, adenylate 3′ Ends, ligate adapters, enrich DNA fragments, purity and validate library. TruSeq Stranded Total RNA with Ribo-Zero Gold (RS-122-2301, Illumina) was used in these processes, according to the manufacturer’s instructions. The cDNA library was constructed and sequenced on the Illumina Hiseq X Ten, PE150 platform at Shanghai Ouyi Biomedical Technology co., Ltd (Shanghai, China). The sequencing depth was 12 G clean data.

For the bioinformatics analysis, SortMeRNA v4.2.0 (Cedex, Paris, France) and Trimmomatic v0.36 (Julich, Germany) were used to remove residual rRNA sequences and low-quality reads, respectively, resulting in high-quality clean reads. Hisat2 v2.0.4 (Baltimore, MA, USA) was used to align the high-quality clean reads to the reference genome of the mice. The samples were evaluated by genomic and gene alignment. Stringtie v1.3.1 (Baltimore, MA, USA) was used to assemble the reads and splice new transcripts. Fragments per kilobase of transcript per million mapped reads (FPKM) were obtained by Cuffdiff software (Massachusetts, USA) to determine the level of transcript expression. Expression abundances were log2-transformed to normalize the expression levels per transcript. Gene Ontology (GO) and Kyoto Encyclopedia of Genes and Genomes (KEGG) pathway analysis were performed with functional annotation of the differentially expressed genes.

### 2.9. Tandem Mass Tag (TMT) Mass Spectrometry Analysis

Frozen colon tissues were treated with a buffer (7 mM sucrose, 1 mM NaCl, 0.39 mM EDTA·2 Na, 0.086 mM DTT) and supplemented with 1 mM PMSF. Then, the samples were homogenized by sonication and added to Tris-phenol buffer and mixed for 30 min at 4 °C. The mixtures were centrifuged at 7100× *g* for 10 min at 4 °C to obtain the supernatants. Afterwards, the supernatants were precipitated for 5 times with the volumes of 0.1 M cold ammonium acetate-methanol buffer and kept at −20 °C overnight. After precipitation, the proteins were washed with cold methanol and acetone successively. Finally, the extracted protein concentrations were determined by BCA assay (cat. no. 23227, Pierce, Thermo Scientific, Landsmeer, Netherlands) and stored at −80 °C until use.

After protein quantification, the samples were added to sequencing-grade trypsin. Next, peptides from each sample were lobed with TMTpro label reagent (Thermo, Waltham, MA, USA). The resulting peptides were analyzed with Agilent 1100 HPLC (Agilent, Santa Clara, CA, USA) with an Agilent Zorbax Extend C column (5 μm, 150 mm × 2.1 mm). The mobile phases A and B were 2% acetonitrile in water (pH = 10) and 98% acetonitrile in water (pH = 10), respectively. The peptides were separated as follows: 0~8 min, 98% A; 8~8.01 min, 98%~95% A; 8.01~48 min, 95%~75% A; 48~60 min, 75~60% A; 60~60.01 min, 60~10% A; 60.01~70 min, 10% A; 70~70.01 min, 10~98% A and 70.01~75 min, 98% A. The collected samples were used for subsequent liquid chromatography with tandem mass spectrometry (LC-MS/MS) analysis.

The LC-MS/MS analysis was performed with the EASY-nLCTM 1200 system (Thermo, Waltham, MA, USA). The peptides were loaded in a capillary trap column (100 μm × 2 cm, RP-C18, Thermo Fisher) and then separated by a capillary analytical column (15 cm × 75 μm, RP-C18, Thermo Fisher). The samples were eluted with the following gradients: 0~50 min, 5–28% B; 50~60 min, 28–42% B; 60~65 min, 42–90% B and 65~75 min, 90% B.

All raw data were analyzed by Proteome Discover 2.4 (Thermo, Waltham, MA, USA). Mass tolerances for precursor and fragment ions were 0.02 Da and 10 ppm, respectively. The false discovery rate (FDR) was less than 0.01 and the number of peptides had to be over 2 for further quantification. Protein–protein interaction (PPI) was used to analyze the differentially expressed proteins based on STRING database. Cytoscape v3.8.0 (Bethesda, Maryland, USA) software was used to visualize the PPI network.

### 2.10. Correlations Analysis of Proteome and Transcriptome

To evaluate the correlation between transcriptome and proteome levels in the colon, Pearson correlation coefficients were performed. A correlation heat map was presented by GraphPad Prism version 8.0 (GraphPad Software Inc., San Diego, CA, USA).

### 2.11. Real-Time PCR

To verify the results of the transcriptome, the extracted RNA from the colon was reverse transcribed into cDNA, using the PrimeScript RT Master Mix kit (cat. no. RR036a, TaKaRa, Shiga, Japan). Then, the cDNA was performed on the QuantstudioTM 7 flex system (ABI Q7 Flex 384 well, Life Technologies, San Diego, CA, USA) with the TB Green® Premix Ex Taq ™ II (cat. no. RR820a, TaKaRa, Tokyo, Japan) and gene-specific primers (Table S1). The relative expression was calculated by the 2-ΔΔCt formula, where ∆∆Ct = (CtTarget-Ct GAPDH) DSS+mEVs-(CtTarget-Ct GAPDH) DSS+PBS. GAPDH was used as the house-keeping gene for normalization.

### 2.12. Statistical Analysis

All data were presented as the mean ± SEM and drawn by GraphPad Prism 8.0 (GraphPad Software, Inc. San Diego, CA, USA). The comparation of body weights, DAI, colonic length and histological score of colons were performed by one-way ANOVA analysis, and relative mRNA expression obtained from real-time PCR was analyzed by unpaired Student’s *t*-test of SPSS version 22.0 (SPSS, lnc., Chicago, IL, USA). A significant difference was declared at *p* < 0.05. The principal component analysis (PCA) was performed using R Script. For RNA sequencing data, the genes were filtered according to the value of counts, and only those with a value greater than 2 were analyzed further. DESeq2 v1.14 (New York, NY, USA) software was used to standardize the counts number of each sample gene, and calculate the foldchange (FC). Then, the NB (negative binomial distribution test) was used as the significance test. Only FC > 1.5/FC < 0.67 and FDR < 0.05 were considered statistically significant. For the proteomic data, the protein abundance was analyzed by Student’s *t*-test with *p* < 0.05 and FC ≥ 1.2/FC ≤ 0.84 being considered statistically significant.

## 3. Results

### 3.1. Characterization of Bovine mEVs

**Figure 1:**
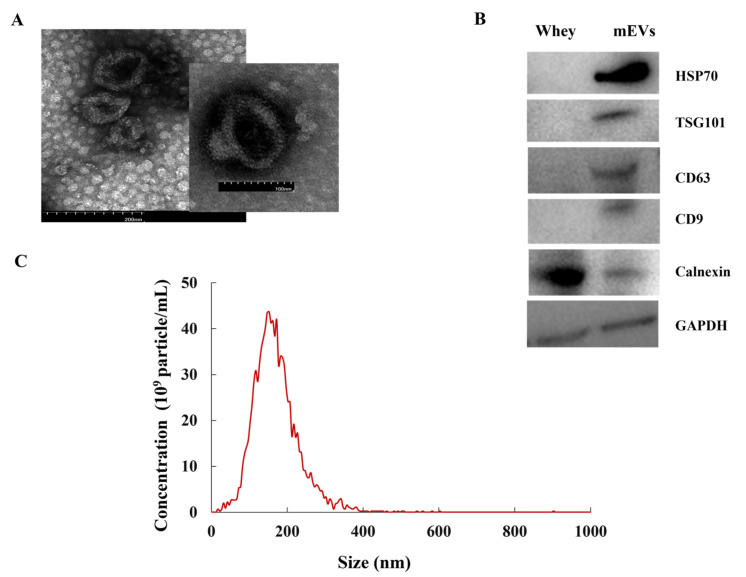
The characterization of mEVs. (**A**) The image of mEVs by transmission electron microscopy. (**B**) The whey and mEVs samples characterized by Western blot for the presence of markers HSP70, TSG101, CD63, and CD9 and negative mEV-marker calnexin. (**C**) The size distribution and concentration analysis of mEVs by nanoparticle tracking analysis.

The mEVs isolated from bovine milk were characterized by TEM, NTA and WB. The shape of the mEVs showed a circular or elliptical structure (Figure 1A). The peak diameter of mEVs was 154.78 ± 5.22 nm (Figure 1C). The mEVs contained abundant positive surface markers HSP70, TSG101, CD63, and CD9 and only a marginal amount of the negative marker calnexin (Figure 1B). The above results confirmed that the isolated particles were mEVs.

### 3.2. Protective Roles of mEVs on DSS-Induced Colitis

**Figure 2:**
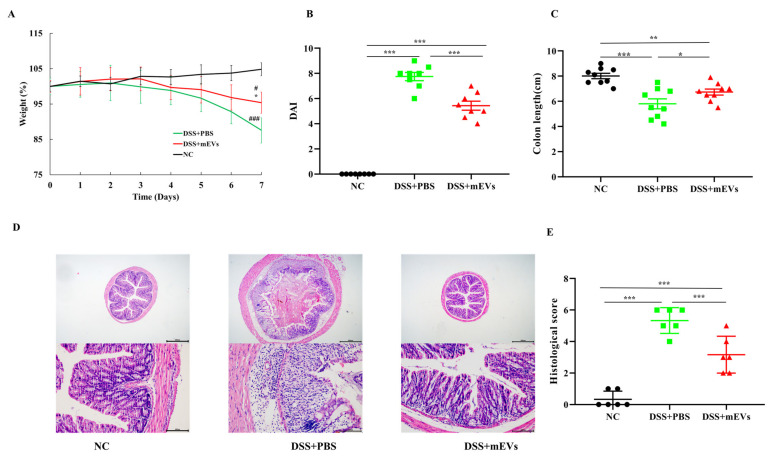
Oral mEVs attenuated the clinical symptoms of DSS-induced experimental colitis in mice. (**A**) Percentage of body weight change. (**B**) Disease activity index (DAI) score for colitis severity. (**C**) The length of colon tissues. (**D**) Representative pictures of hematoxylin and eosin (H&E)-stained colon sections, scale bar = 500 μm (top) and 100 μm (bottom). (**E**) The histology score. Data represent the mean ± SEM, n = 12 samples/group, * *p* < 0.05, ** *p* < 0.01, *** *p* < 0.001. * *p* < 0.05 relative to DSS+PBS group; # *p* < 0.05, ### *p* < 0.001 relative to NC group.

Compared with the DSS+PBS group, mEV intervention significantly attenuated DSS-induced colitis, as evidenced by the significantly reduced body weight loss, relieved DAI score, and decreased colon shortening (*p* < 0.05) (Figure 2A–C). Investigation of colonic morphology presented that oral mEVs significantly reduced the infiltration of inflammatory cells, mucosal damage and overall histology score, compared with the DSS+PBS group (Figure 2D,E). These results suggested that pre-supplementation of mEVs was capable of suppressing DSS-treated colitis symptoms and colonic injury.

### 3.3. mEVs Changed Gut Gene Expression Profile and Signaling Pathways

To explore the mechanisms associated with the phenotype changes, we investigated the colonic transcriptome differences between the DSS+PBS and DSS+mEVs groups. A total of 1,289,007,754 raw reads (193.34 Gb) and 1,263,042,544 (185.56 Gb) clean reads were produced. The Q30 of all the reads ranged from 90.75% to 93.30% and the average GC content was 48.41% (Table S2). The average percent of reads that were consistently mapped to the transcriptome was 95.90%. The FPKM and gene expression density are shown in Figure S1, presenting comprehensive representation of the transcriptome (Figure S1A,B).

**Figure 3:**
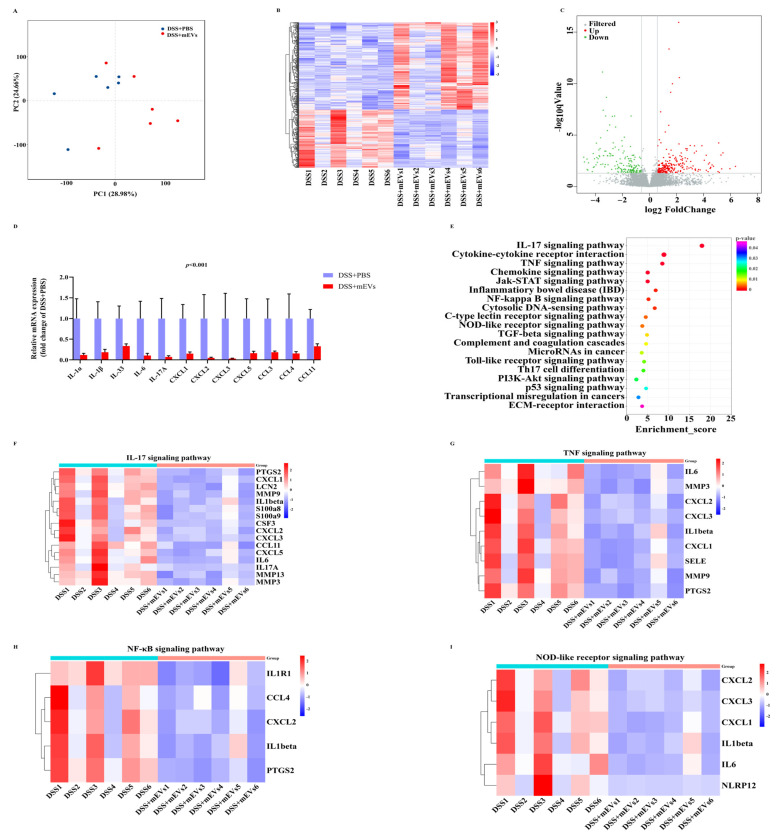
mEVs altered colonic gene expression in DSS-induced colitis. (**A**) Principle component analysis (PCA) of the gene expression profiling in the colonic tissues. (**B**) Heatmap of hierarchical clustering for the transcriptional profiling in the 12 colonic tissues. (**C**) Volcano plot of the alteration of colonic genes in response to mEVs (FC > 1.5/FC < 0.67, FDR < 0.05). (**D**) The relative abundance levels of partial inflammatory factors according to transcriptomic analysis. (**E**) KEGG pathway enrichment analysis of the most significantly down-regulated pathways. (**F**–**I**) Heatmap of the inflammation-related pathways enriched in the significantly down-regulated genes.

mEVs significantly regulated the colonic gene expression in the DSS model, as evidenced by the PCA and hierarchical clustering based on Pearson’s correlation coefficients in all samples (Figure 3A,B). Compared with the DSS+PBS group, oral mEV intervention significantly up-regulated the expression of 246 genes (FC > 1.5 and *p* < 0.05) and down-regulated the expression of 164 genes (FC < 0.67 and *p* < 0.05) (Figure 3C, Table S3). In the presence of mEVs, the expression of proinflammatory cytokines (*IL-1α*, *IL-1β*, *IL-33*, *IL-6* and *IL-17A*) and cytokine receptors (*IL-1R1* and *IL-1R2*) were significantly decreased in DSS-treated mice (*p* < 0.05) (Figure 3D, Table S3). Among these genes, *IL-1β*, *IL-6* and *IL-17A* have critical roles in the onset and progression of IBD [21]. In addition, mEV administration significantly inhibited the expression of chemokine ligands (*CXCL1*, *CXCL2*, *CXCL3*, *CXCL5*, *CCL3*, *CCL4* and *CCL11*) (*p* < 0.05), chemokine receptors (*CXCR2* and *CCR3*), growth factors (*CSF3*), extracellular matrix remodeling enzymes (*MMP3*, *MMP8*, *MMP9*, *MMP10*, *MMP12*, *MMP13*, *PLAUR* and *TIMP1*), oxidative stress (*PTGS2*), pro-inflammatory mediators (*S100a8*) and other candidate biomarkers (*PROK2*) (Figure 3D, Table S3), in comparison with the DSS+PBS group. To verify the results of the RNA-seq analysis, a total of six differentially expressed genes in this study were selected to further investigate their expression profiles (Figure S2). Taken together, the reduction in inflammatory cytokines and chemokines levels in colon tissues suggested that mEVs exhibited preventive and therapeutic effects in colitis mice.

The changes in GO terms and KEGG pathways in response to mEV supplementation were also examined. According to the GO analysis of differentially expressed genes, extracellular region, extracellular space, inflammatory response, neutrophil chemotaxis and cytokine activity were the top five significantly down-regulated GO terms (*p* < 0.05) (Figure S1D). In addition, circadian regulation of gene expression, rhythmic process, Z disc, circadian rhythm and membrane were the top five significantly up-regulated GO terms (*p* < 0.05) (Figure S1E). The KEGG annotation analysis of differentially expressed genes revealed that 44 pathways were significantly down−regulated (Figure 3E) and 13 pathways were significantly up-regulated (*p* < 0.05) (Figure S1C). The down-regulated pathways were associated with inflammation, such as the IL-17 signaling pathway, TNF signaling pathway, NF-κB signaling pathway and NOD-like receptor signaling pathway (Figure 3F–I). In addition, the IBD pathway was also down-regulated (Figure 3E). Moreover, the up-regulated KEGG pathways were closely associated with the metabolism of xenobiotics by cytochrome P450, glycosaminoglycan biosynthesis-keratan sulfate and steroid hormone biosynthesis. These data demonstrated that mEVs ameliorated colitis through modulating multiple pathways, including inflammation−related signaling pathways and nutrition metabolism.

### 3.4. mEV Supplementation Altered the Colonic Proteome

**Figure 4:**
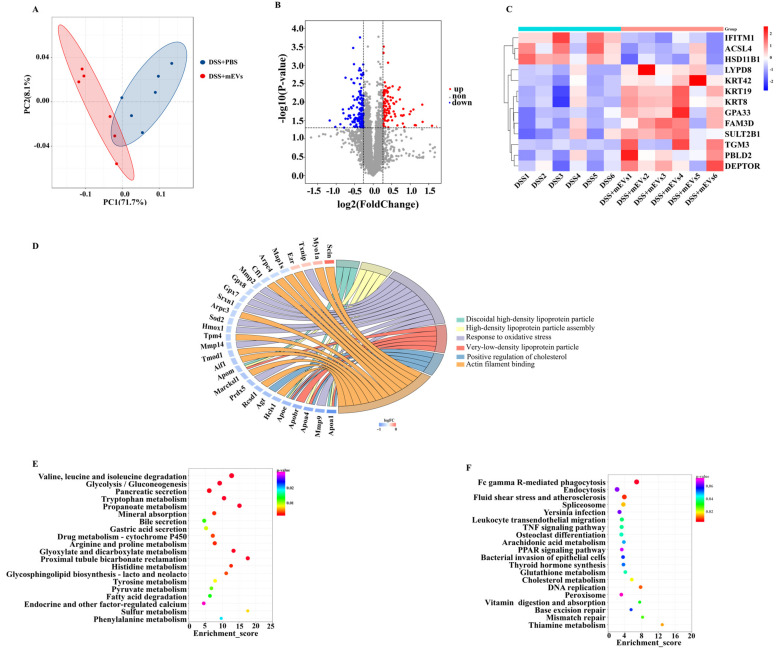
Tandem mass tag (TMT) mass spectrometry of the distinct proteomic signatures of colons in DSS−induced colitis murine model. (**A**) Principle component analysis (PCA) of the protein expression profiling in DSS+PBS and mEV−fed group samples. (**B**) Volcano plots of the alteration in colonic proteins in response to mEVs (FC ≥ 1.2/FC ≤ 0.84, *p* < 0.05). (**C**) The heatmap of the 13 differently expressed proteins. (**D**) The enrichment chord plot of differentially expressed proteins (the number of differentially expressed proteins in every sample were more than 3 and less than 500. (**E**) The up−regulated KEGG pathways in response to mEVs. (**F**) The down−regulated KEGG pathways in response to mEVs.

Colon tissue proteome profiling discovered 86,396 effective spectrum numbers, 48,164 peptides and 5322 proteins. PCA revealed the significant separation in proteomes between mEVs-fed and DSS+PBS groups (Figure 4A). Among these proteins, 109 proteins were up-regulated and 150 proteins were down-regulated in the colons of mEV-fed mice versus DSS-treated mice (FC ≥ 1.2/FC ≤ 0.84 and *p* < 0.05) (Figure 4B, Table S4). Furthermore, Figure 4C presented typical differentially expressed proteins, including the following nine up-regulated proteins: protein-glutamine gamma-glutamyltransferase E (TGM3), Ly6/PLAUR domain-containing protein 8 (LYPD8), phenazine biosynthesis-like domain-containing protein 2 (PBLD2), protein FAM3D, keratin, type II cytoskeletal 8 (KRT8), sulfotransferase 2B1 (SULT2B1), cell surface A33 antigen (GPA33) and DEP domain-containing mTOR-interacting protein (DEPTOR), and the following three down-regulated proteins: long-chain-fatty-acid--CoA ligase 4 (ACSL4), interferon-induced transmembrane protein 1 (IFITM1) and corticosteroid 11-beta-dehydrogenase isozyme 1 (HSD11B1). Furthermore, to verify the validity of the proteome results by WB, we randomly selected four differentially expressed proteins (SCIN, ACSL4, ABAT and DDC). The results of WB were consistent with the proteome analysis (Figure S3).

The 56 significantly enriched GO terms, based on the enrichment scores of the differentially expressed proteins in DSS-treated mice with and without receiving oral mEVs, in the cellular component, biological process and molecular function were listed in Table S5 (*p* < 0.05). The top three most enriched pathways were discoidal high-density lipoprotein particle, glutamine-fructose-6-phosphate transaminase (isomerizing) activity and CoA carboxylase activity. Further enrichment chord analysis showed that discoidal high-density lipoprotein particle, high-density lipoprotein particle assembly, response to oxidative stress, very low-density lipoprotein particle, positive regulation of cholesterol esterification and actin filament binding were involved in the attenuation of colitis by oral mEVs (Figure 4D).

To further understand the cellular pathways through which the annotated unigenes were potentially related to ripening, the 259 differentially expressed proteins were mapped to the KEGG database. In total, 23 KEGG pathways were significantly up-regulated and 14 were significantly down-regulated. Oral mEVs significantly improved amino acid metabolism and lipid metabolism, including valine, leucine and isoleucine degradation, tryptophan metabolism, arginine and proline metabolism, histidine metabolism, pyruvate metabolism and fatty acid degradation (Figure 4E). Moreover, Fc gamma R-mediated phagocytosis, leukocyte transendothelial migration and TNF signaling pathways were the main down-regulated KEGG pathways in response to oral mEVs (Figure 4F). These results suggested that mEVs-induced metabolic reprogramming of amino acid metabolism and lipid metabolism in the colon was beneficial for protecting against colitis.

### 3.5. Correlation Analysis of mRNAs and Proteins

**Figure 5:**
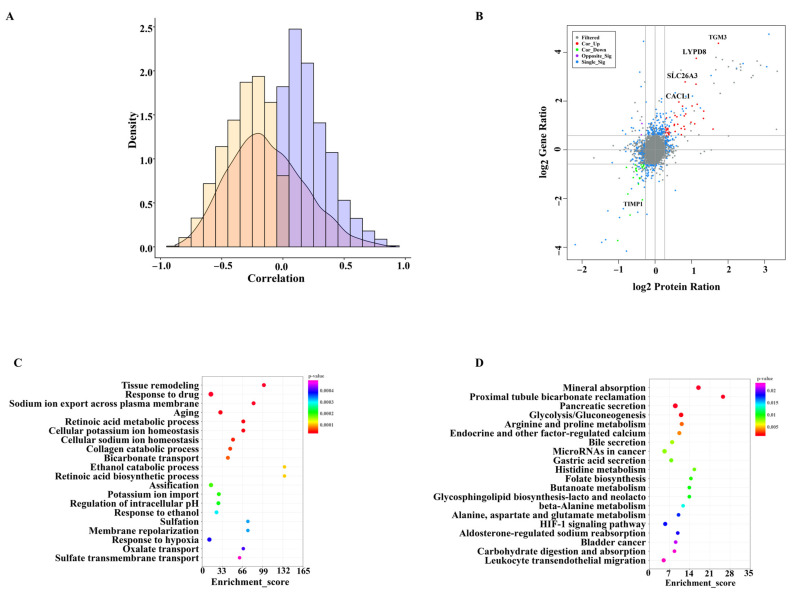
Correlation analysis of mRNA and protein levels. (**A**) The combination results of deferentially expressed genes and proteins. (**B**) Distribution of Spearman correlation coefficients of mRNA−to−protein abundance in the colon. (**C**) GO terms in the biological process category of the differentially expressed mRNA−proteins. (**D**) KEGG pathway analysis of differentially expressed mRNA−proteins.

We explored the correlation between the transcriptome and proteome and found that the regulation of gene abundance resulted in a weaker tendency for changes in the corresponding protein level (Figure 5A). A total of 60 proteins and genes with same changes were identified from the differentially expressed proteins and genes (Figure 5B). Among them, 39 proteins−genes were co-upregulated (such as TGM3, CLCA1, SLC26A3, and LYPD8), and 21 were co-downregulated (Figure 5B). GO and KEGG enrichment analyses were used to explore the potential functions of the 60 same changes in the proteins and genes. “Tissue remodeling” and “metabolism” were the main terms that were enriched in the biological process category (Figure 5C). In addition, the KEGG pathway analysis showed that the genes were enriched in metabolic pathways and many signaling pathways, including histidine metabolism, arginine and proline metabolism, alanine, aspartate and glutamate metabolism, carbohydrate digestion and absorption and fatty acid degradation (Figure 5D).

## 4. Discussion

mEVs have been proved to transfer from milk into the plasma through the intestinal tract of the consuming organism, and to affect gene expression once absorbed by target cells, thereby interfering with the physiological and pathological processes of the consumers [22,23,24]. Reif et al. reported that mEVs could be absorbed by intestine cells, exerting a therapeutic and anti-inflammatory role in DSS−induced colitis, which involves reducing the expression of *IL-6* and *TNF-α* [25]. In another study, mEVs also had anti−inflammatory capacity by inhibiting the expression of the pro-inflammatory cytokines *IL-6* [26]. Benmoussa et al. further determined the concentration of cytokines, chemokines and inflammation−related proteins in the colon of colitis mice, and demonstrated that mEVs decreased the production of the intestinal inflammation driver and pro-inflammatory cytokines [27]. Consistent with the above studies, we observed the protective and therapeutic roles of mEVs on DSS−induced murine colitis, as evidenced by a decrease in body weight loss, DAI score, colonic shortening and histology score. We also found that mEVs decreased the expression of pro-inflammatory factors (*IL-1β*, *IL-6* and *IL-17A*) in colitis. Therefore, nucleic acids, proteins and lipids delivered by mEVs into recipient cells changed their biological responses, and effectively attenuated the symptoms of acute colitis.

Chemokines are a subset of cytokines, which could induce immune cell migration to sites of inflammation, ultimately resulting in tissue damage and destruction [28]. *CCL11*, also known as eotaxin-1, is considered as an eosinophil chemoattractant, and is involved in the pathogenesis of DSS−induced colitis [29]. *CCL3* and *CCL4*, known as *MIP-1α* and *MIP-1β*, respectively, are indispensable mediators of polymorphonuclear leukocyte and macrophage recruitment. In addition, *CCL3* can specifically move macrophages and granulocytes to the inflammatory area in acute inflammation [28]. Members of the *CXCL* chemokine families (e.g., *CXCL1, CXCL2*, *CXCL3* and *CXCL5*) are also closely related with immune response. *CXCL1* is also known as *NAP3*, which induces neutrophil migration [30]. *CXCL2* is also known as *MIP2-α*, which is secreted by monocytes and macrophages [30]. Hence, the anti-inflammatory mechanisms of mEVs are mainly associated with its ability to inhibit chemokine expression.

Apart from the above chemokines’ genes, mEVs also changed other genes’ expression, when compared to the DSS+PBS group. *CYP1A1* is reported to modulate immune responses in the intestine and exert protection against intestinal inflammation [31,32]. The *SLC6A4* gene is significantly down-regulated during intestinal inflammation, which codes for a protein that is the only high affinity and competent transporter of serotonin [33]. *SLC6A8*, also known as *CRT*, is the only famous creatine transporter, and might regulate epithelial integrity and barrier function [34]. Hence, loss of *SLC6A8* expression could induce metabolic disorders and can usually be found in IBD patients [34]. *PHLPP2* might be a promising candidate therapeutic target for colitis, and the up-expression of *PHLPP2* could inhibit the NF-κB signaling pathway to alleviate colonic inflammatory response [35,36]. *ANO1* is vital for the protection of intestinal epithelium from colitis, whereas its expression was significantly decreased in the DSS-induced colitis mice [37,38]. *NR1D1* knockout mice were more susceptible to experimental colitis, suggesting an essential role of *NR1D1* in colitis development [39,40]. Moreover, *NR1D1*, as a transcriptional repressor, could repress the NF-κB/Nlrp3 axis to prevent colitis [39]. Although *CPA6* is closely associated with the extracellular matrix or immune response, its expression was decreased in colitis [41]. *GLP-2R* has the ability to improve crypt cell proliferation, suppress epithelial cell apoptosis and maintain mucosal integrity. However, the level of GLP-2R was reduced in colitis in both mice and patients [42]. *GSTT1* is found to alleviate colitis by the IL-22 dependent pathway [43]. *SlCO2A1* deficiency is identified to deteriorate intestinal inflammation by activating the *NLRP3* inflammasome in macrophages [44]. For the reasons above, the up-regulated *CYP1A1*, *SLC6A4*, *SLC6A8*, *PHLPP2*, *ANO1*, *NR1D1*, *CPA6*, *GLP-2R*, *GSTT1* and *SlCO2A1* in mEV−fed mice indicated that mEVs facilitated the expression of genes that had low expression in colitis, and hence ameliorated colitis.

Additionally, the results of the proteomic changes could provide further mechanistic understanding of how mEVs alleviate acute colitis. A previous study demonstrated that the expression of PBLD was significantly decreased in mice with DSS-induced colitis; however, its repressive action on NF-κB signaling could improve intestinal barrier function and alleviate intestinal inflammation [45]. FAM3D, as a protector of colonic homeostasis, is reported to maintain the normal differentiation of goblet cells, as well as the presence of acidic mucins and antimicrobial molecules [46]. The KRT8 protein plays an important role in anti-inflammatory processes and in sustaining cellular structural integrity [47]. SULT2B1 is a critical enzyme that catalyzes the synthesis of cholesterol sulfate from cholesterol, and its deficiency promotes pro-inflammatory macrophage polarization [48]. Protein GPA33 is a marker of Treg cells, and GPA33-deficient mice would increase the rate of colitis [49]. DEPTOR has the ability to inhibit the activity of mTOR, which could promote inflammation by secreting cytokines (TNF-α, IL-1β, and IL-6) and chemokines (CCL7 and CXCL16) [50]. ACSL4, an important enzyme of long-chain fatty acid degradation, is involved in inflammasome activation in neutrophils, and is significantly up-regulated in inflamed colon tissues [51]. The polymorphisms of IFITM1 increase the sensitivity to ulcerative colitis, and the expression of IFITM1 is also up-regulated in colitis [52]. HSD11B1 exerts an important role in the inflammatory response, and the high expression of HSD11B1 will increase the incidence of colitis [53]. Therefore, the increased levels of PBLD, FAM3D, KRT8, SULT2B1, GPA33, DEPTOR, and decreased expression of ACSL4, IFITM1 and HSD11B1 caused by mEVs has been demonstrated to attenuate colitis, implying that consumption of mEVs has the potential to improve intestinal health.

A previous study found that the correlation coefficients between mRNAs and protein levels are often low [54]. Our study also revealed the poor correlation between transcriptional activity and protein production. The poor correlation may be explained by the post-transcriptional regulation and different half-life of mRNAs and proteins [55]. Although our results showed a non-perfect correlation between the transcriptomic and proteomic surveys, there were some similar influences on several genes/proteins. In our study, the expression of *TGM3*, *CLCA1*, *SLC26A3*, and *LYPD8* genes/proteins were significantly up-regulated in response to mEVs (Figure 5B). *TGM3* is expressed and synthesized in goblet cells, which exclusively determines the transamination activity of colonic mucus [56]. *CLCA1* also maintains mucus homeostasis to decrease the risk of colitis [57]. *SLC26A3* contributes to protecting the intestinal epithelial barrier and maintaining the enterocyte acid/base balance. Thus, the high expression of *SLC26A3* could prevent DSS−induced acute colitis [58]. *LYPD8* levels are significantly decreased in colitis patients, and supplementation of LYPD8 proteins might maintain colonic homeostasis in colitis patients by inhibiting the motility of pathogenic bacteria, such as γ-proteobacteria [59,60].

Overall, the altered gene and protein expressions derived from mEV intervention might play a central role in the attenuation of colitis, which could also be used as novel candidate biomarkers for the treatment of intestinal disorders. However, this study did not exclude the effects of slight impurities from the remaining milk ingredients. Furthermore, adding a mEV−depleted bovine milk group would offer more convincing evidence.

## 5. Conclusions

Bovine mEVs attenuated DSS−induced acute colitis models, as evidenced by increased body weight, reduced DAI and maintained integrity of the colon tissue. Transcriptome data revealed that mEVs inhibited the expression of pro-inflammatory cytokines (*IL-1β* and *IL-6*) and chemokines (*CXCL1*, *CXCL2* and *CXCL3*) and promoted the expression of anti-inflammatory genes. Moreover, this study provided evidence of the proteomic variations in the colon in response to oral mEVs. By integrating the results of transcriptomics and proteomics, we found that amino acid metabolism and immune-related signaling pathways played a crucial role in the process of mEVs in alleviating colitis. Overall, mEV supplementation in the current study presented great potential and many desirable features for the prevention of acute colitis.

## Data Availability

All the raw sequences of the transcriptome were submitted to the NCBI Sequence Read Archive (SRA), under accession number PRJNA821425S.

## Supplementary Materials

The following supporting information can be downloaded at: https://www.mdpi.com/article/10.3390/nu14153057/s1, Table S1. The list of primer sequences. Table S2. The results of raw reads after quality control. Table S3. Quantitative analyses of transcriptomic data at the gene level. Table S4. Quantitative analyses of proteomic data. Table S5. GO analysis of differently expressed proteins in response to mEVs. Figure S1. (A) The expression of normalized genes (FPKM) in 12 colonic tissues. (B) Distribution of transcript abundance in 12 colonic tissues. (C) The up-regulated KEGG pathways in the transcriptome. (D) The down-regulated GO terms in the transcriptome. (E) The up-regulated GO enrichment in the transcriptome. Figure S2. The results of real-time PCR. Figure S3. Western blotting of differentially expressed proteins.
