# Supplementary material for: Effects of Milk-Derived Extracellular Vesicles on the Colonic Transcriptome and Proteome in Murine Model
Source: Nutrients. 2022 Jul 26;14(15):3057. doi: 10.3390/nu14153057 (PMC9332160; doi:10.3390/nu14153057)
Supplement: Supplementary file 1 [file nutrients-14-03057-s001.zip › Supplementary Tables S1-S2.pdf]

Supplementary tables

Table S1 The list of primer sequences.

Genes	Forward	Reverse
IL-1β	5'-CTTTCCCGTGGACCTTCCAG-3'	5'-ATGGGAACGTCACACACCAG-3'
IL-6	5'-GGCGTTTGGGTTGCTTCTCTGT-3'	5'-GTGGAGGAGAGGTCGTCTTGCT-3'
CXCL1	5'-CGCCTATCGCCAATGAGCTG-3'	5'-GACTTCGGTTTGGGTGCAGT-3'
CXCL2	5'-CACCAACCACCAGGCTACAG-3'	5'-TTTGACCGCCCTTGAGAGTG-3'
CXCL3	5'-CCATCCAGAGCTTGACGGTG-3'	5'-TGGGGGTTGAGGCAAACTTC-3'
CXCL5	5'-TCCTCAGTCATAGCCGCAAC-3'	5'-ATGACTTCCACCGTAGGGCA-3'
GAPDH	5'-TCAAGCTCATTTCCTGGTATGAC -3'	5'-GGATAGGGCCTCTCTTGCTC-3'

Table S2 The results of raw reads after quality control.

Sample	Raw Reads	Raw Bases	Clean Reads	Clean Bases	Q30	GC
DSS1	115430150	17314522500	113165272	16609775892	92.47%	49.07%
DSS2	108079222	16211883300	105961128	15549272743	92.81%	48.24%
DSS3	103622982	15543447300	101790180	14962216665	93.30%	48.19%
DSS4	100936008	15140401200	98754824	14503433026	92.33%	48.17%
DSS5	103422984	15513447600	101109714	14891815698	92.65%	48.12%
DSS6	103859934	15578990100	101672186	14937526802	93.03%	48.66%
DSS+mEVs1	117158740	17573811000	114738168	16908658773	92.67%	48.30%
DSS+mEVs2	117901596	17685239400	115459844	16983991091	93.17%	48.32%
DSS+mEVs3	102074758	15311213700	99996516	14662777611	92.73%	48.42%
DSS+mEVs4	107387930	16108189500	105206816	15467333086	93.03%	48.85%
DSS+mEVs5	102624438	15393665700	100720102	14784428593	93.21%	48.18%
DSS+mEVs6	106509012	15976351800	104467794	15314626997	90.75%	48.40%
