# Supplementary figures and images for: Effects of Milk-Derived Extracellular Vesicles on the Colonic Transcriptome and Proteome in Murine Model
Source: Nutrients. 2022 Jul 26;14(15):3057. doi: 10.3390/nu14153057 (PMC9332160; doi:10.3390/nu14153057)

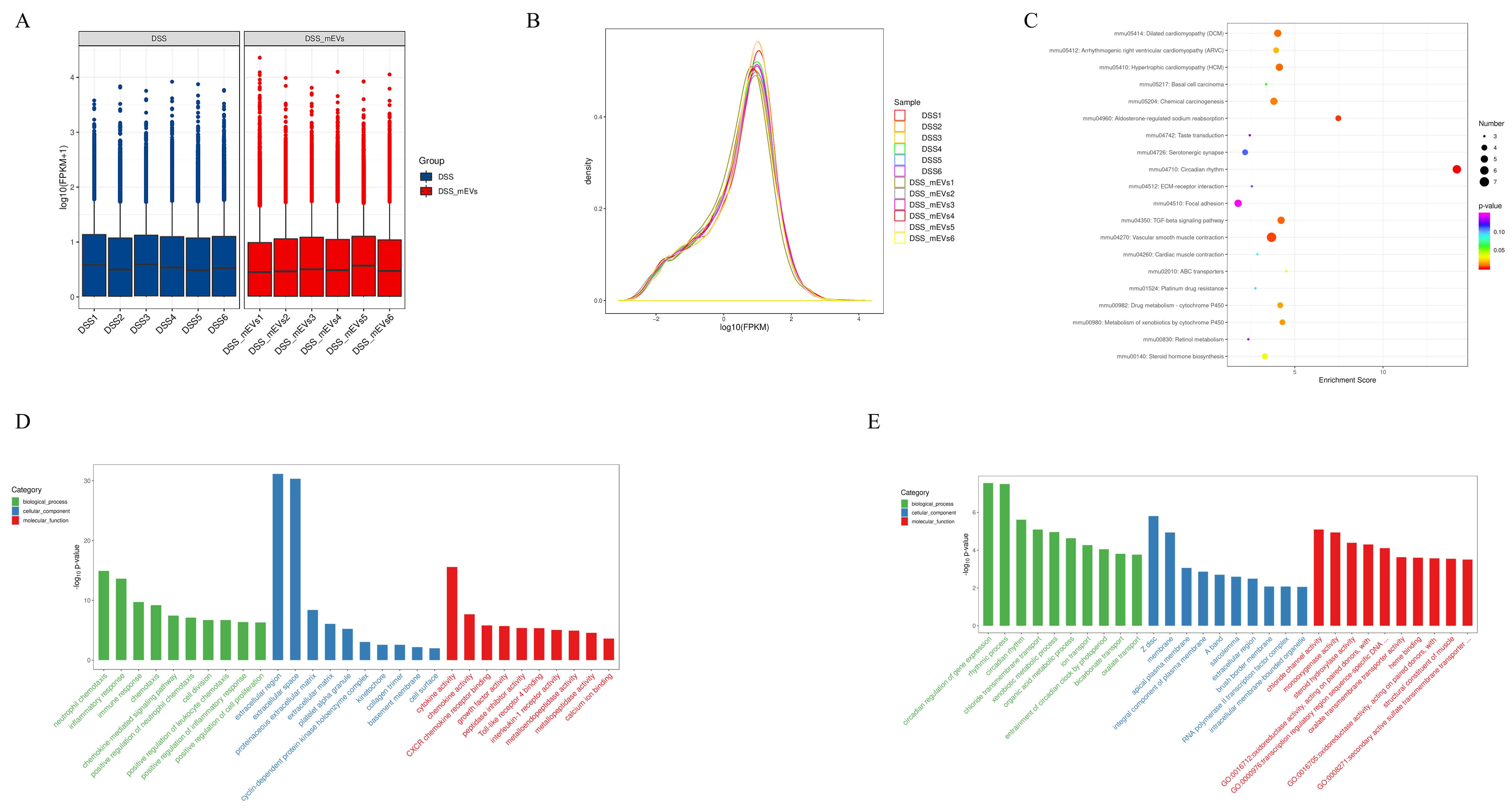

Supplement: Supplementary file 1 [file nutrients-14-03057-s001.zip › Figure S1.tif]

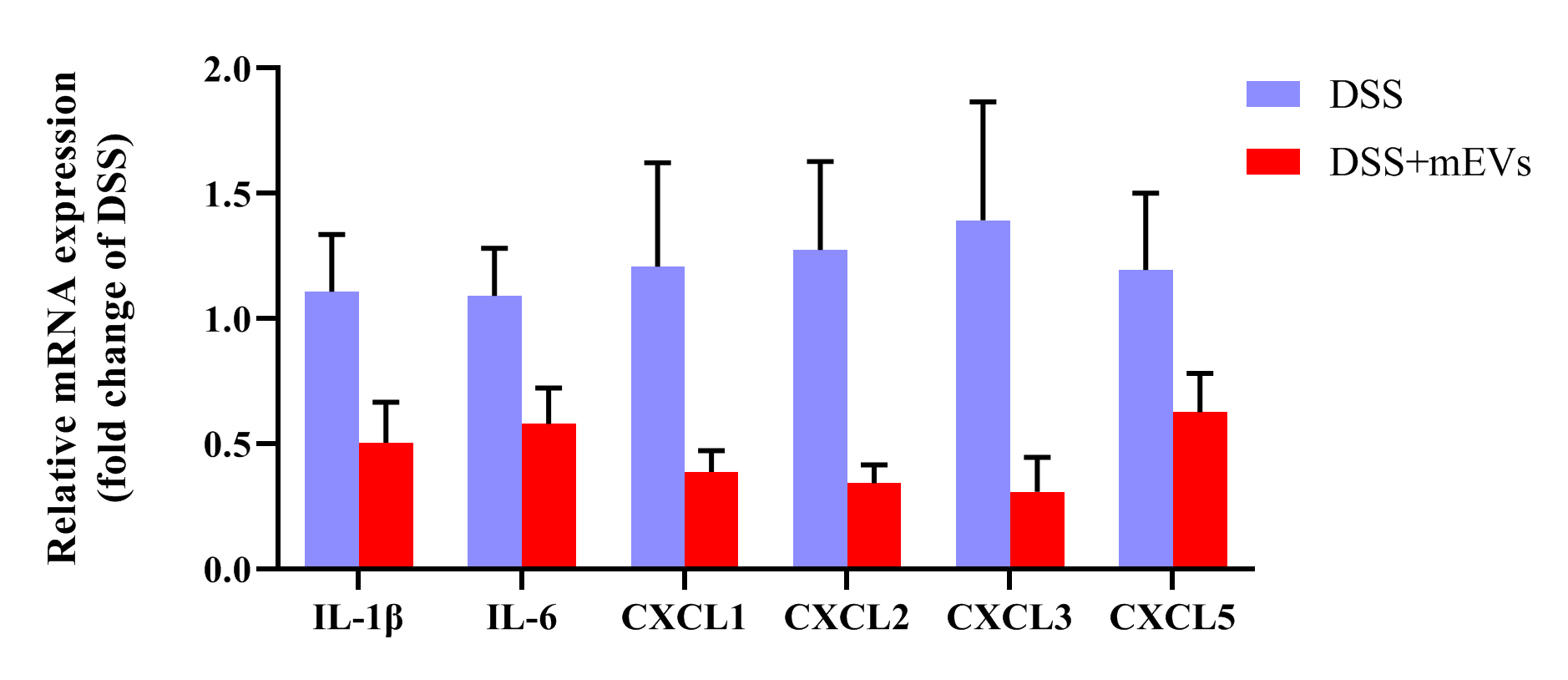

Supplement: Supplementary file 1 [file nutrients-14-03057-s001.zip › Figure S2.tif]

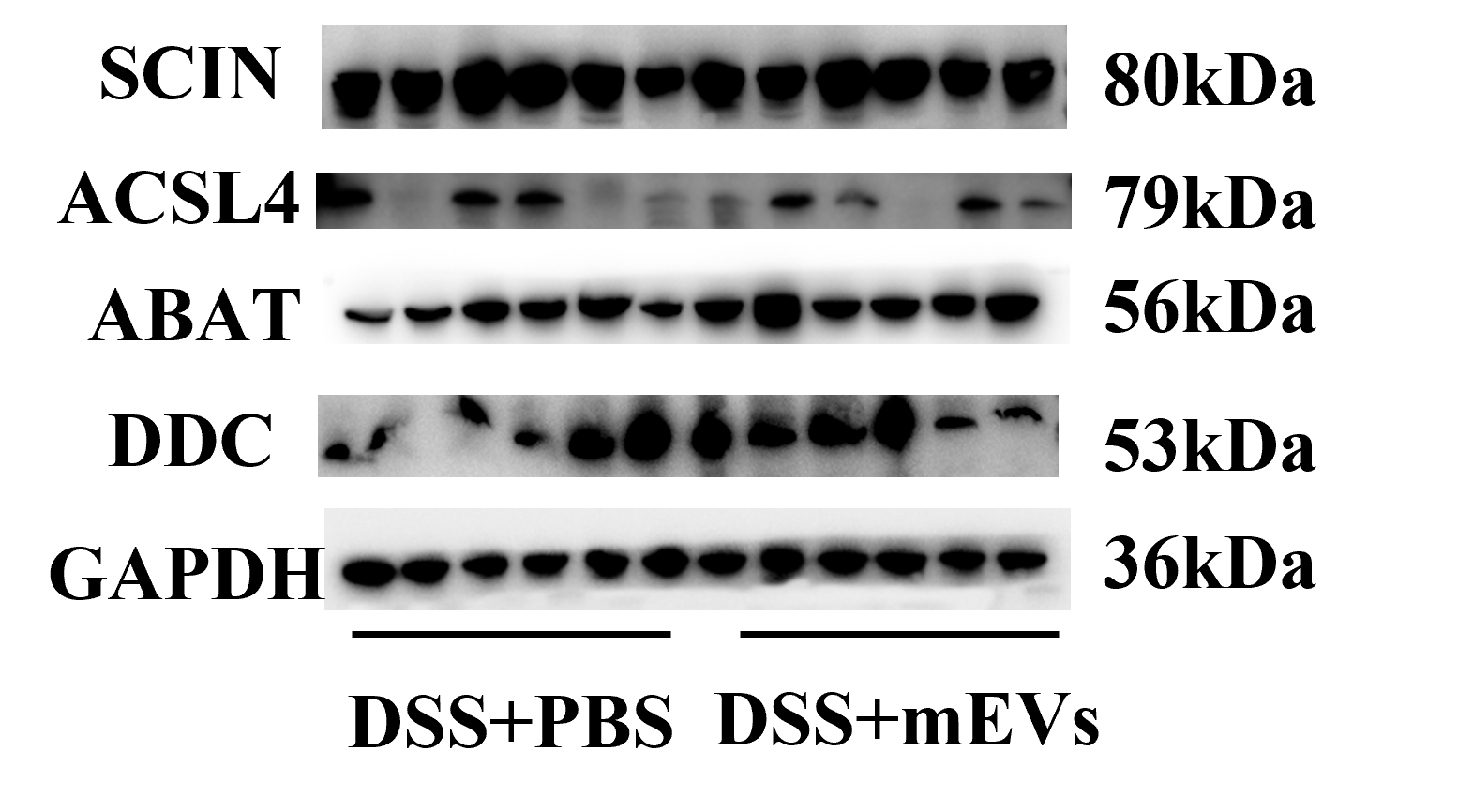

Supplement: Supplementary file 1 [file nutrients-14-03057-s001.zip › Figure S3.tif]
